# Unraveling the impact of *trip12* on neurodevelopment: insights from a zebrafish model

**DOI:** 10.1093/braincomms/fcag276

**Published:** 2026-07-15

**Authors:** Maider Roibás-Santos, Paula Suárez-Bregua, Josep Rotllant, Ángel Carracedo, Catarina Allegue, Laura Sánchez, Andrés Blanco-Hortas, Alba Pensado-López

**Affiliations:** Department of Zoology, Genetics and Physical Anthropology, Faculty of Veterinary, Universidad de Santiago de Compostela, Lugo 27002, Spain; Aquatic Biotechnology Lab, Instituto de Investigaciones Marinas, Consejo Superior de Investigaciones Científicas (IIM-CSIC), Vigo 36208, Spain; Centro Oceanográﬁco de Vigo, Instituto Español de Oceanografía (IEO-CSIC), Vigo 36390, Spain; Aquatic Biotechnology Lab, Instituto de Investigaciones Marinas, Consejo Superior de Investigaciones Científicas (IIM-CSIC), Vigo 36208, Spain; Genomics and Bioinformatics Group, Centro de Investigación en Medicina Molecular y Enfermedades Crónicas (CiMUS), Universidad de Santiago de Compostela, Santiago de Compostela 15782, Spain; Genetics Group, Instituto de Investigación Sanitaria de Santiago (IDIS), Santiago de Compostela 15706, Spain; Medicine Genomics Group, Centro de Investigación Biomédica en Red de Enfermedades Raras (CIBERER), Instituto de Salud Carlos III, Madrid 28029, Spain; Fundación Pública Galega de Medicina Xenómica (FPGMX), Servizo Galego de Saúde (SERGAS), Santiago de Compostela 15706, Spain; Genomics and Bioinformatics Group, Centro de Investigación en Medicina Molecular y Enfermedades Crónicas (CiMUS), Universidad de Santiago de Compostela, Santiago de Compostela 15782, Spain; Genetics Group, Instituto de Investigación Sanitaria de Santiago (IDIS), Santiago de Compostela 15706, Spain; Medicine Genomics Group, Centro de Investigación Biomédica en Red de Enfermedades Raras (CIBERER), Instituto de Salud Carlos III, Madrid 28029, Spain; Fundación Pública Galega de Medicina Xenómica (FPGMX), Servizo Galego de Saúde (SERGAS), Santiago de Compostela 15706, Spain; Department of Zoology, Genetics and Physical Anthropology, Faculty of Veterinary, Universidad de Santiago de Compostela, Lugo 27002, Spain; Department of Zoology, Genetics and Physical Anthropology, Faculty of Veterinary, Universidad de Santiago de Compostela, Lugo 27002, Spain; Medical Oncology Unit, Fundación Pública Galega de Investigación Biomédica (INIBIC), Complexo Hospitalario Universitario de A Coruña (CHUAC), A Coruña 15006, Spain

**Keywords:** *Danio rerio*, CRISPR/Cas9, neural development, cognitive impairment, RNAseq

## Abstract

Thyroid Hormone Receptor Interactor 12 (TRIP12) is an E3 ubiquitin ligase capable of mediating ubiquitin-dependent proteolysis of specific protein substrates. This function regulates key biological processes, including cell cycle progression, cell differentiation, chromatin remodelling and DNA damage repair. Consequently, loss-of-function mutations in *TRIP12* have been associated with a broad spectrum of human diseases, including cancer and neurological and neurodevelopmental disorders. Previous studies have demonstrated that pathological variants of *TRIP12* cause Clark–Baraitser syndrome, characterized by craniofacial dysmorphism, motor delay and intellectual disability, with or without autism spectrum disorder. Despite the well-characterized clinical manifestations, the underlying molecular pathways affected by *TRIP12* disruption and their implication in the pathophysiology of autism spectrum disorder and intellectual disability remain unclear. Using a knock-out zebrafish model, we have elucidated the essential role of *trip12* in diverse metabolic and biological pathways, particularly those related to neural and neurodevelopmental processes, shedding light on potential mechanisms underlying the pathogenesis. Heterozygous and recessive homozygous zebrafish mutants exhibit clinical features analogous to those observed in human patients, including craniofacial anomalies and decreased locomotor activity. Furthermore, this study provides substantial evidence for the vital role of *trip12* in the early stages of development, as homozygous individuals exhibited early mortality by Day 23 post-fertilization, while a substantial mortality rate of 90% was observed by Day 35 in ‘heterozygous’ mutants. The present study demonstrates the profound impact that *trip12* mutations have on embryogenesis, and transcriptomic analysis offers an in-depth knowledge of the molecular basis of the disease. These findings offer valuable insights into potential therapeutic targets for improving outcomes in individuals with *TRIP12*-associated disorders.

## Teaser

Roibás-Santos *et al*. explored, in a zebrafish model, how variations in *TRIP12*, an intellectual disability-related gene, lead to the pathogenesis of the disease. Features observed in patients (cranial and motor abnormalities) were recapitulated, and several dysregulated pathways were unravelled, shedding light on potential therapies.

## Graphical Abstract

**Graphical Abstract:**
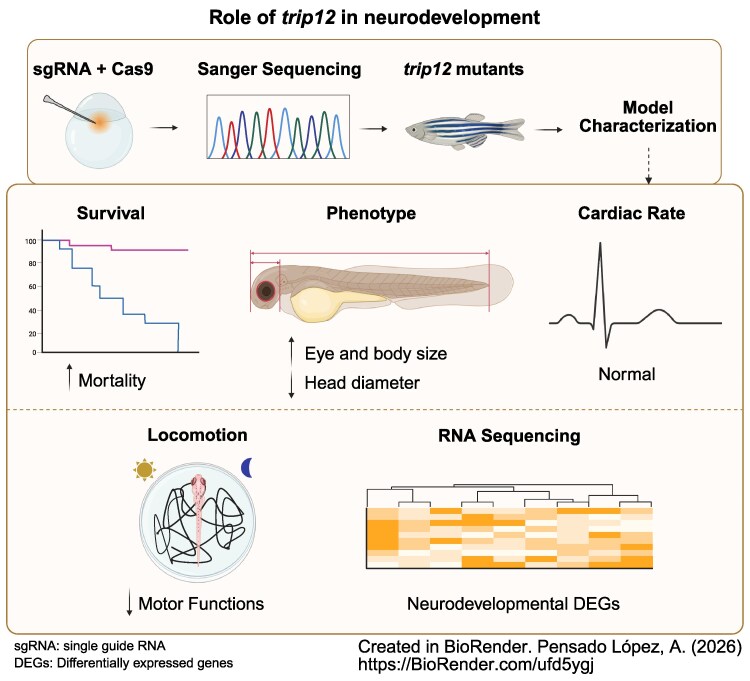

## Introduction

Thyroid Hormone Receptor Interactor 12 (TRIP12) is a HECT (Homologous to the E6-AP Carboxyl Terminus) domain-containing E3 ubiquitin ligase capable of mediating ubiquitin-dependent proteolysis of specific protein substrates. This function allows it to regulate key biological processes, including cell cycle progression, cell differentiation, chromatin remodelling and DNA damage repair.^1,2^ Due to these major functions, pathogenic TRIP12 variants have been associated with a diverse spectrum of human disorders, including cancer and various forms of neurological and neurodevelopmental disorders.^1,3,4^

In particular, *TRIP12* has been identified as a primary gene for autism spectrum disorder (ASD) and intellectual disability (ID),^5^ and it has been cited as a causative gene for Clark–Baraitser syndrome. This rare autosomal dominant neurodevelopmental disorder is characterized by severe cognitive impairment, craniofacial dysmorphism and behavioural manifestations, among other symptoms.^6^ Other clinical findings related to mutations in *TRIP12* include growth restriction, obesity, motor delay or seizures and recognizable facial features, such as deep-set eyes, a broad nasal tip, a wide mouth and low-set ears.^7^ Pathogenic variants responsible for this condition include deletions, duplications or substitutions leading to premature stop codons, frameshift mutations, missense changes or the complete absence of protein production. A notable observation is that the severity of the phenotype does not appear to be influenced by the type or location of the variant.^3,5,7^

The *TRIP12* gene (GRCh38.p14, Chromosome 2: 229,763,837-229,923,239, ENSG00000153827.15) encodes 30 splice variants, 19 of which are protein-coding (Gene: TRIP12 (ENSG00000153827) - Summary - Homo_sapiens - Ensembl genome browser 113).^8^ The most conserved and expressed transcript (NM_001348323.3, ENST00000675903.1) includes 42 exons and encodes for a 2067 amino acid protein consisting of four domains: (i) HECT domain contains the ubiquitin-conjugating E2 enzyme and the catalytic cysteine residue, which is essential for ubiquitin ligase catalytic activity^9,10^; (ii) tryptophan-tryptophan-glutamate (WWE) domain required for TRIP12 interaction with some of its substrates, such as PTF1a or APP-BP1^11-13^; (iii) armadillo repeats (ARM), also required for protein–protein interactions^14^; and (iv) intrinsically disordered regions (IDR) domain responsible for the interaction of TRIP12 with chromatin, thereby modifying chromatin structure and regulating gene expression.^15^ The amino acid sequence and domains of TRIP12 are highly conserved throughout evolution, especially in vertebrates, with ∼82 and 98% identity for zebrafish and mouse, respectively. This high degree of conservation suggests essential functions for the protein in living organisms.^1^ In fact, the major role of TRIP12 has been evidenced by the embryonic lethality of a murine model carrying an inactivating mutation in *TRIP12.*^16^ Nevertheless, the molecular pathways affected as a consequence of *TRIP12* disruption and their implication in the pathophysiology of ASD and ID are not yet fully understood.

Zebrafish (*Danio rerio*) is a widely used animal model for studying several human pathological processes, especially neurodevelopmental^17^ and neurodegenerative diseases.^18^ Aside from these ease of manipulation and rapid development of zebrafish, among other advantages, the most important brain regions and major subdivisions, as well as cell types, differentiation mechanisms, connectivity patterns, signalling pathways and gene expression profiles, are highly conserved.^19,20^ Given these facts, together with the ∼83% homology between the human *TRIP12* gene and the zebrafish *trip12* gene, we performed loss-of-function studies using the zebrafish model to explore the role of *trip12* in neurodevelopment. We generated a CRISPR/Cas9 *trip12* zebrafish mutant line with the aim of exploring its impact on embryogenesis, phenotype and behaviour and, more specifically, describing the molecular mechanisms underlying the disease. Our findings confirm and broaden the preliminary results found in our previously generated *trip12* knockdown zebrafish model (see Supplementary material).

## Materials and methods

### Zebrafish husbandry

Wildtype (WT) zebrafish of the AB strain were maintained in the fish facilities of the Department of Zoology, Genetics and Physical Anthropology at the University of Santiago de Compostela (AE-LU-003). The water temperature was maintained at 28°C, pH ∼7 and conductivity of ∼600 µS. The fish were kept on a 14 h light and 10 h darkness photoperiod, following established protocols.^21,22^ All animal care, maintenance and experimental procedures were performed in accordance with current guidelines from the European Community and Spanish Government on animal care and experimentation (Directive 2010-63-UE and RD 53/2013). The study was approved by the Bioethics Committee of the University de Santiago de Compostela and the Xunta de Galicia government. Randomization and blindness were ensured along the present study as all experimental procedures were performed before the genotyping of animals. The total number of animals used for each of the following experimental procedures (detailed in the figure legends) was obtained from different crosses to ensure biological variability.

### CRISPR/Cas9-mediated knockout of *trip12* and genotype characterization

A *trip12* loss-of-function mutation was generated using the CRISPR-Cas9 gene editing system. The CRISPR protocol was adapted from one kindly provided by Dra. Fernández Miñán (Andalusian Center for Developmental Biology, CABD). The target region was the first coding exon of the *trip12* gene. A gene-specific oligonucleotide (5′- aattaatacgactcactataGGACGGCGCAGTGACCCCCCgttttagagctagaaatagc-3′) was designed using the CRISPRscan web tool.^23^

This oligonucleotide was amplified by PCR using a common scaffold oligonucleotide (5′-GATCCGCACCGACTCGGTGCCACTTTTTCAAGTTGATAACGGACTAGCCTTATTTTAACTTGCTATTTCTAGCTCTAAAAC-3′) to obtain a 125-base pair (bp) fragment, using the iProof™ High-Fidelity DNA Polymerase (Bio-Rad), following the manufacturer’s instructions. The PCR product was then transcribed using the MAXIscript™ T7 Transcription Kit (Thermo Fisher Scientific) and purified with the RNA Clean & Concentrator-5 Kit (Zymo Research). The concentration of the resulting guide RNA (gRNA) was measured using a NanoDrop® 2000 spectrophotometer (Thermo Fisher Scientific). One-cell stage zebrafish embryos were microinjected with 2–4 nl of a mixture containing 15–40 ng/μl of the gRNA, 1.2 μg/μl of TrueCut™ Cas9 Protein v2 (Thermo Fisher Scientific) and 0.1% phenol red solution.

The efficiency of the CRISPR/Cas9 protocol and the generation of mutations were verified by detecting heteroduplexes on a 5% polyacrylamide gel. Genomic DNA was isolated from injected embryos at 48 h post-fertilization (hpf) using Chelex-100 resin (Bio-Rad) and amplified by PCR with AmpliTaq Gold™ DNA Polymerase (Thermo Fisher Scientific) and the following primers: forward 5′-GAGGATGAGGGAACATCCAGA-3′ and reverse 5′-CTGCTGCACAAACGGAACTG-3′. After confirming mutations, F0 embryos were raised to adulthood. Founder mutants, identified by the presence of heteroduplexes, were outcrossed with WT individuals. The presence of mutations in F1 embryos and F1 adults was verified by heteroduplex analysis. Mutations were characterized by TA-cloning (Original TA Cloning Kit, Invitrogen) and Sanger sequencing (BigDye Terminator v3.1 Cycle Sequencing Kit, Applied Biosystems), following the manufacturer’s indications. Sequences were analysed using the CodonCode Aligner software, and *in silico* analysis of the resulting proteins from mutated sequences was performed with the ExPASy-Translate Tool.^24^

Heterozygous adult animals (*trip12* +/−) from the F1 generation carrying the same mutation were in-crossed to produce the F2 generation (25% +/+, 50% +/−, 25% −/−), which was used for subsequent experiments. Because a homozygous stable line could not be generated due to the lethality of homozygous mutants within the first 23 days post-fertilization (dpf), the genotype of each fish was analysed by agarose gel electrophoresis and fragment analysis (GeneScan 500 LIZ dye, Applied Biosystems) at the end of each experiment. A schematic representation of the CRISPR/Cas9 protocol and genotype characterization is shown in Supplementary Figs 1 and 2.

### Survival analysis

In the absence of adult homozygous mutants, a survival analysis was conducted to determine the developmental stage at which homozygosity for the *trip12* mutation resulted in lethality. To this end, several crosses between WT individuals (namely, +/+ pure) or heterozygous mutants were performed, and egg fertilization was confirmed. The development and mortality of the offspring were assessed twice daily. Dead embryos or larvae were collected and immediately frozen in Chelex-100 for later genotyping. At 35 dpf, the remaining larvae were genotyped by caudal fin-clipping, and the resulting data were analysed as described below.

### Phenotype characterization

Embryos from the F2 generation were used for phenotypic characterization at 48 hpf. Embryos were anaesthetized with 0.02% tricaine methanesulfonate (MS-222, Sigma-Aldrich), and images were captured using a Nikon Ds-Ri1 camera attached to an inverted fluorescence microscope (AZ100 Multizoom, Nikon). Images were analysed using Nis-Elements BR (Version 4.13.04 64-bit) to measure three phenotypic features in each embryo: body length, head diameter and eye area. The resulting data were exported to Microsoft Excel and analysed as described below.

### Mutant’s cardiac rate

To detect potential cardiac abnormalities in *trip12* mutant zebrafish, the heart rate of embryos was analysed at 3 dpf, based on previous studies.^25,26^ Larvae were anaesthetized with 0.01% tricaine and positioned laterally on a microscope slide. Heartbeat was recorded and measured for 20 s using a Nikon microscope connected to Viewpoint Heart-Beat/Blow-Flow software (Viewpoint Life Sciences). The resulting data were exported to Microsoft Excel and analysed as described below.

### Larval locomotor activity

General motor function was evaluated in larvae at 5 and 7 dpf. In earlier stages, larvae tend to exhibit more explosive movements, while later stages, coinciding with complete yolk reabsorption and the initiation of feeding, may involve altered movement patterns due to foraging behaviour. Larvae were transferred to 24-well plates (one larva per well), and each plate was placed into a Zebrabox device connected to Zebralab software (Viewpoint Life Sciences). Locomotor activity was automatically tracked for 60 min, with alternating 10 min periods of light and dark in a continuous loop. Integrated activity, expressed as the number of pixels moved per minute, was exported to Microsoft Excel and analysed as described below.

### RNA sequencing

For transcriptomic analysis, 48 hpf embryos from the F2 generation were anaesthetized and euthanized with an overdose of tricaine (0.2%). Heads were collected, immersed in RNAlater (Sigma-Aldrich) and stored at −80°C until use. The remaining tissue was used for genotyping. After genotyping, embryos were pooled according to genotype, and RNA was extracted using the miRNeasy Micro Kit (Qiagen). RNA concentration was measured using a NanoDrop® 2000 spectrophotometer (Thermo Fisher Scientific). A total of 9 samples (3 +/+, 3 +/−, 3 −/−), with 1–2 µg of RNA each, were sent for sequencing. RNA integrity was assessed using a 2100 Bioanalyzer (Agilent). All samples had RNA integrity number values ≥4, which is the minimum value for messenger RNA library construction and sequencing. Libraries were sequenced on an Illumina NovaSeq platform (150 bp paired-end reads) by Novogene (Cambridge, UK). The pipeline followed to analyse the RNA sequencing data can be found in GitHub - Roslin-Aquaculture/RNA-Seq-kallisto: Analysis of RNA sequencing Illumina data: differential expression using Kallisto and DESeq2 · GitHub. The quality of the sequencing output was assessed using FastQC v.0.12.1 (Babraham Bioinformatics - FastQC A Quality Control tool for High Throughput Sequence Data).

Low-quality reads, contaminating sequences, low-complexity reads (repetitive DNA sequences) and short reads were removed using Fastp v.0.22.0.^27^ Filtered reads were aligned to the latest version of the zebrafish genome (GRCz11- Danio_rerio - Ensembl genome browser 113), and transcript abundance was quantified using kallisto v.0.46.1.^28^ Computational analysis was performed using the resources of the Supercomputing Centre of Galicia (CESGA).

Lowly expressed genes were filtered before differential expression analysis, retaining only genes with TPM >5 in at least two biological replicates within at least one genotype group. Principal component analysis was used for sample clustering and outlier identification. Differential gene expression analysis among +/+, +/− and −/− individuals was performed using DESeq2 v.1.42.^29^ Differentially expressed genes (DEGs; FDR-adjusted *P*-value < 0.05, |log_2_FoldChange| > 1) were analysed for Gene Ontology (GO) term and Kyoto Encyclopedia of Genes and Genomes (KEGG) pathway enrichment using the Database for Annotation, Visualization and Integrated Discovery (DAVID v2025_1),^30,31^ with the zebrafish transcriptome from this experiment as background. Significantly enriched GO terms and KEGG pathways were identified based on FDR-adjusted *P*-values < 0.05. Plots were generated using the ‘ggplot2’ package.^32^

### Statistical analysis

Data on phenotype, heart rate and locomotion were analysed using GraphPad Prism version 7.02. Statistical comparisons were performed using a one-way ANOVA followed by Dunnett’s multiple comparison test. Survival and RNAseq data were analysed using RStudio v.2023.9.1.494.^33^ For the survival analysis, a Kaplan–Meier survival curve was generated, statistical significance was assessed by pairwise comparisons using the log-rank test and *P*-value was adjusted by Bonferroni correction for multiple comparisons. A detailed description of the RNAseq analysis is provided in the previous section. Statistically significant differences were defined as *P* < 0.05. In the figures, levels of significance are represented as follows: *P < 0.05; **P < 0.01, ***P < 0.001, ****P < 0.0001.

## Results

### CRISPR/Cas9-mediated knockout of *trip12*

**Figure 1:**
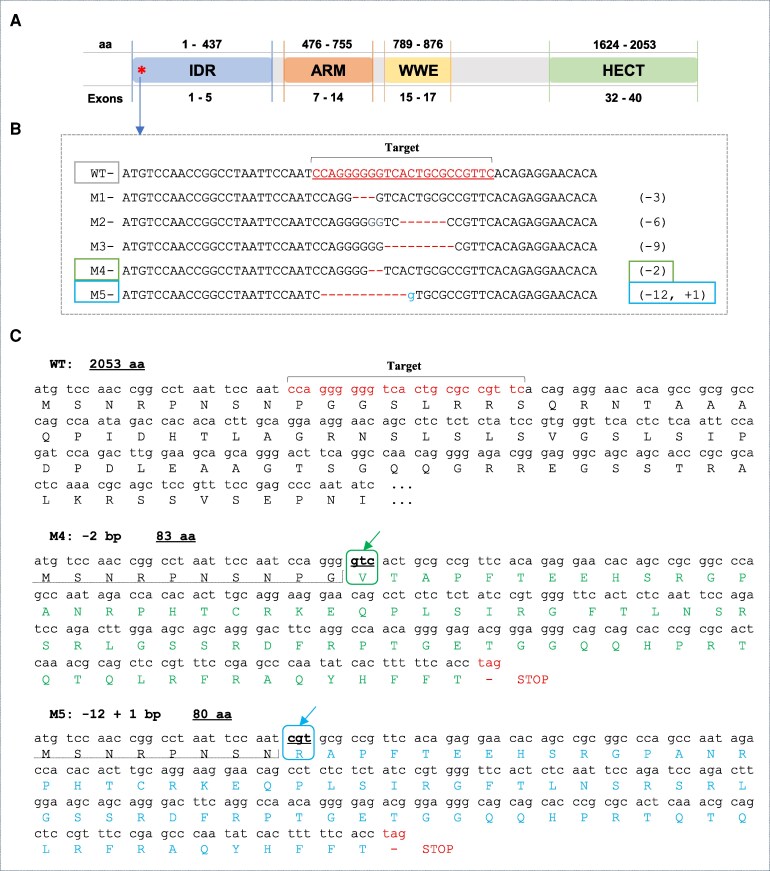
**CRISPR/Cas9 induced mutations in *trip12*.** (**A**) Schematic representation of Trip12 domains and amino acid and exon correspondence, showing the target site (asterisk). (**B**) Sequence of wildtype (WT) *trip12* (first row) and sequences of mutations found in *trip12* (subsequent rows). The number of deleted (−) and inserted (+) bases is described on the right side of each sequence. Mutations of interest are M4; (−2 base pair, bp) and M5 (−12, +1 base pair, bp). (**C**) Predicted amino acid sequence encoded by the WT, M4 mutant and M5 mutant sequences. Arrows indicate amino acid changes induced by M4 and M5 frameshift mutations, respectively. Stop codons are shown as STOP. *N* = 150 individuals (obtained from different biological replicates).

*trip12* mutants were generated using CRISPR/Cas9. The first coding exon was selected as the target site, specifically 24 bp downstream of the ATG start codon (Fig. 1A). Genotype analysis revealed five different mutant alleles (Fig. 1A): M1, M2 and M3, which lacked 3, 6 and 9 bp, respectively, within the target site but conserved the normal open reading frame; and M4 and M5, which were frameshift mutations. The M1, M2 and M3 alleles resulted in the deletion of 1, 2 and 3 amino acids, respectively, compared to the WT protein, and could potentially produce a functional protein. Conversely, *in silico* analysis of the M4 (2 bp deletion) and M5 (12 bp deletion and 1 bp insertion) alleles predicted that they would generate truncated proteins of 83 and 80 amino acids, respectively, compared to the 2053 amino acid WT protein (Fig. 1B). It is notable that both of these truncated proteins lacked most of the IDR domain, a region that has been demonstrated to be essential for the interaction with chromatin,^2^ as well as the ARM and WWE domains, which have been identified as responsible for protein–protein interactions.^12,14,34^ Importantly, the mutated proteins also lacked the C-terminal HECT domain, which is crucial for ubiquitination activity.^9,10^ Since the gross morphology of fish carrying the M4 and M5 mutations was similar, and because heterozygous individuals carrying the M5 allele could be distinguished by agarose gel electrophoresis, we chose to proceed with the analysis of this mutation.

### Survival analysis

**Figure 2:**
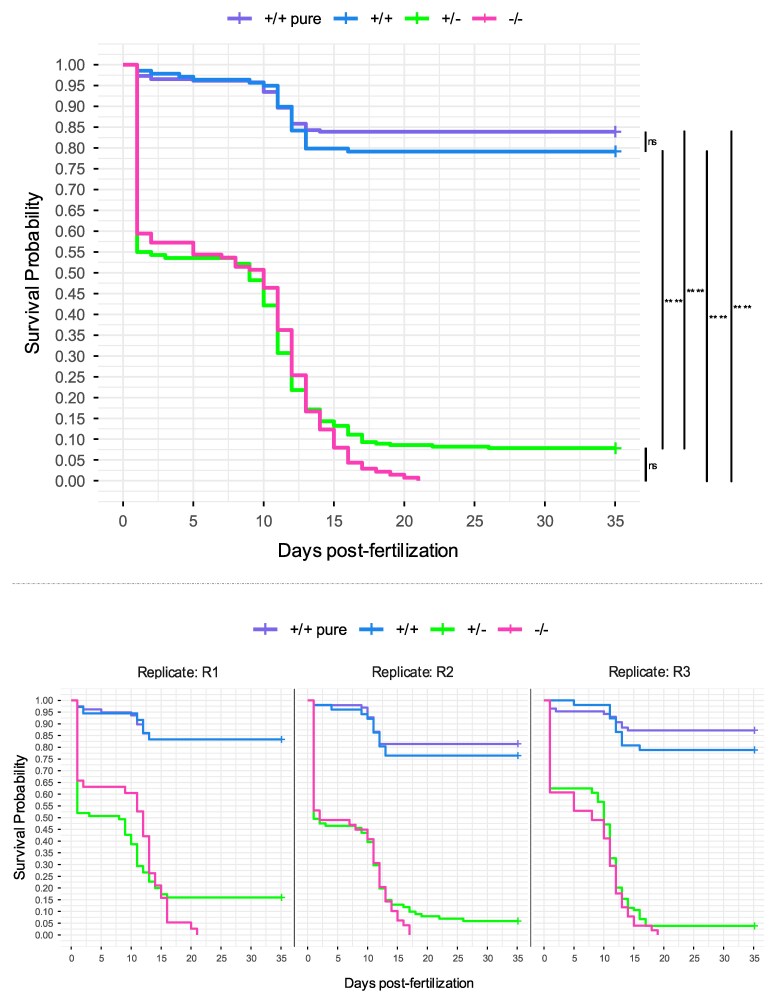
**Survival analysis.** Kaplan–Meier curve comparing survival of wildtype (WT) individuals obtained from WT × WT crosses (+/+ pure) and WT (+/+), *trip12* heterozygous (*+/−*) mutants and *trip12* homozygous (−/−) mutants obtained from *+/−* × *+/−* crosses. Statistical significance was assessed by pairwise comparisons using the log-rank test followed by Bonferroni’s multiple comparisons test. *P < 0.05, **P < 0.01, ***P < 0.001, ****P < 0.0001. *N* = 261 (+/+ pure; R1: 78, R2: 97, R3:86), *N* = 139 (*trip12* +/+ ; R1: 36, R2: 51, R3: 52), *N* = 280 (*trip12* +/−; R1: 75, R2: 101, R3: 104), *N* = 138 (*trip12* −/−; R1: 38, R2: 49, R3: 51).

As shown in Fig. 2, both *trip12 +/−* and *trip12* −/− mutants exhibited decreased survival probability, reaching 50% survival probability at around 10 days. By the end of the 35-day analysis, survival probabilities had further decreased to 8% for *trip12 +/−* and 0% for *trip12* −/, since *trip12* −/− mutants did not survive beyond 21 dpf. The log-rank test with Bonferroni correction revealed significant differences in survival between *+/+* pure or *trip12 +/+* and *trip12 +/−* (*P* < 0.0001) and between *+/+* pure or *trip12 +/+* and *trip12* −/− (*P* < 0.0001) individuals, while the comparison between *+/+* pure and *trip12 +/+* and *trip12 +/−* and *trip12* −/− was not significant (*P* = 1).

### Phenotype analysis of *trip12* mutants

**Figure 3:**
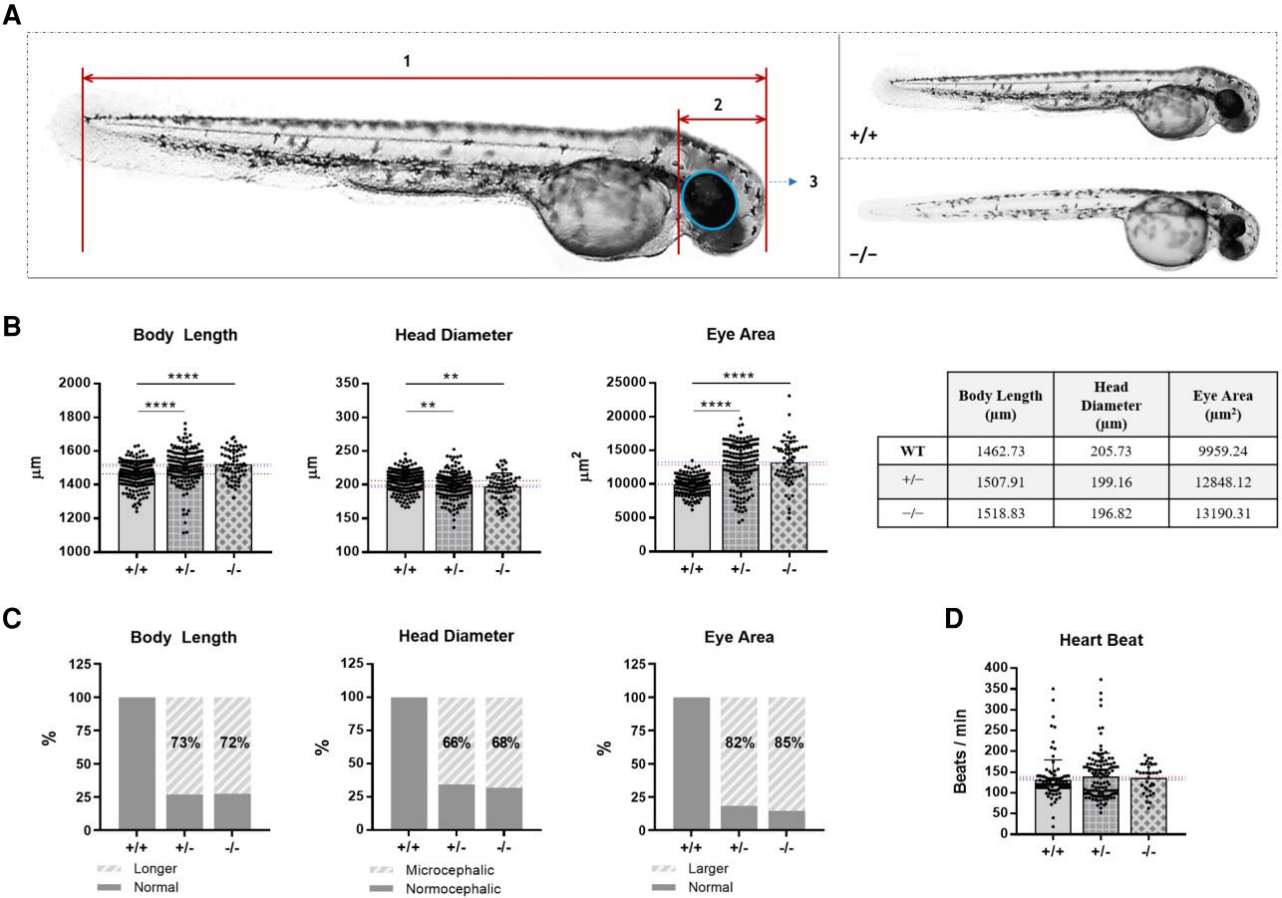
**Phenotypic and cardiac rate analysis of *trip12* mutants.** (**A**) Schematic representation of measurements taken in 48 hpf wildtype (WT) and *trip12* mutants for body length (1), head diameter (2) and eye area (3) (left panel). Representative images of WT (+/+) and *trip12* homozygous (−/−) mutant (right panel). (**B**) Comparison of body length, head diameter and eye area among WT and heterozygous (+/−) and homozygous mutants (left panels). Detailed values are shown in the table (right panel). (**C**) Percentage of heterozygous and homozygous individuals with increased body length, microcephaly and larger eye area. (**D**) Comparison of the heart rate, in terms of beats per minute, for WT and *trip12* mutants. Each dot represents individual larvae. Statistical analysis was performed using one-way ANOVA followed by Dunnett’s multiple comparisons test. *P < 0.05, **P < 0.01, ***P < 0.001, ****P < 0.0001. For phenotype characterization, *N* = 196 (+/+), *N* = 164(+/−), *N* = 68 (−/−). For cardiac rate analysis, *N* = 95 (+/+), *N* = 118 (+/−), *N* = 35 (−/−).

To identify potential morphological abnormalities, embryos at 48 hpf were imaged and analysed. Although the gross morphology of mutant embryos appeared normal compared to WT individuals (Fig. 3A), measurements of body length, head diameter and eye area revealed statistically significant differences. As shown in Fig. 3B, both heterozygous and homozygous mutants had increased body length (+/+: 1462.73 µm; +/−: 1507.91 µm; −/−: 1518.83 µm) and eye area (+/+: 9959.24 µm^2^; +/−: 12 848.12 µm^2^; −/−: 13 190.31 µm^2^), while head diameter was decreased in *trip12* mutants (+/+: 205.73 µm; +/−: 199.16 µm; −/−: 196.82 µm). Overall, 73% of heterozygous mutants and 72% of homozygous mutants had increased body length (Fig. 3C, left panel), 66% of heterozygous mutants and 68% of homozygous mutants had decreased head diameter (Fig. 3C, central panel), and 85% of heterozygous mutants and 82% of homozygous mutants had increased eye diameter (Fig. 3C, right panel). Similarly, *trip12* morphants at 48 hpf displayed an abnormal phenotype compared to their WT siblings (Supplementary Fig. 3A and B).

### Cardiac rate

Decreased survival in heterozygous individuals, lethality in homozygous mutants and the association of cardiac failure with decreased survival in ID patients^35^ led us to evaluate cardiac activity in *trip12* mutant zebrafish. In addition, TRIP12 is known to act on SOX6, which is crucial for the maintenance of cardiac cells,^36^ and *SOX6* has been found to be upregulated in some patients with *TRIP12* mutations.^37^ Cardiac function was assessed by measuring heart rate. We found that neither heterozygous nor homozygous mutants showed significant differences in heart rate compared to WT individuals (Fig. 3D). Additionally, the gross morphology of the heart was normal in mutants. These results suggest that the lethality of homozygous mutants is not related to cardiac abnormalities, at least in terms of heart rate.

### Locomotor analysis of *trip12* mutants

**Figure 4:**
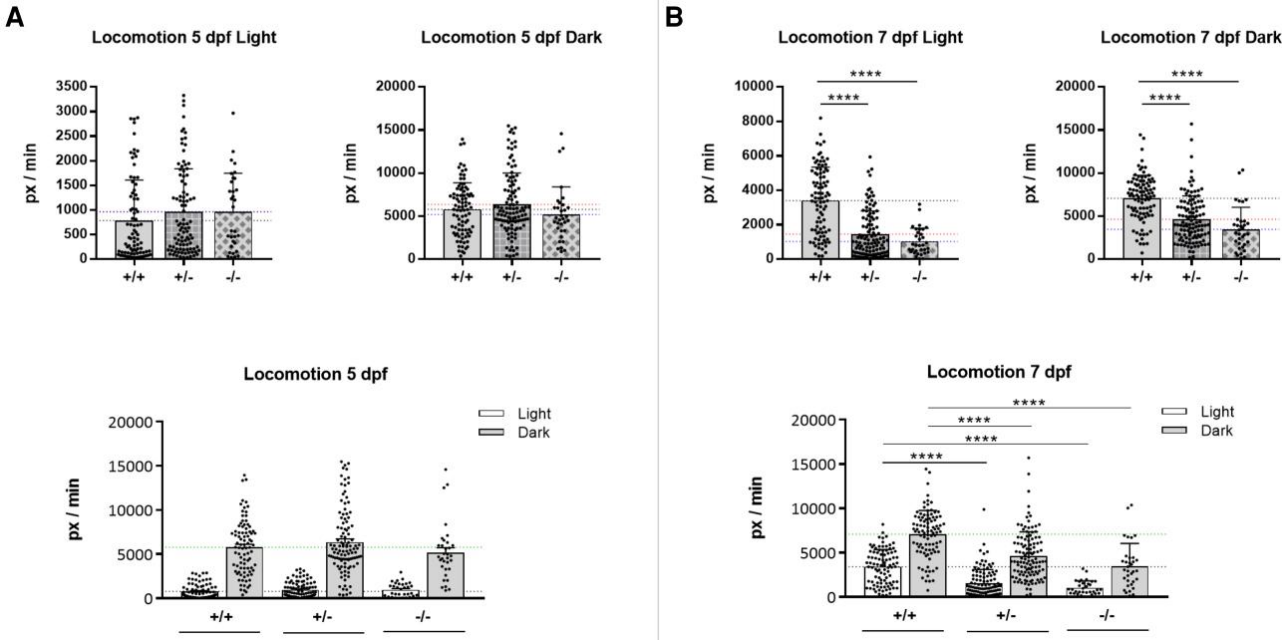
**Locomotor analysis of *trip12* mutants.** Total number of pixels moved per minute in light and dark conditions for 5 dpf (**A**) and 7 dpf (**B**) wildtype (WT, +/+), *trip12* heterozygous (+/−) and homozygous (−/−) individuals, represented separately by condition (*top* panels) and altogether (*bottom* panels). Each dot represents individual larvae. Statistical analysis was performed using one-way ANOVA followed by Dunnett’s multiple comparisons test. *P < 0.05, **P < 0.01, ***P < 0.001, ****P < 0.0001. *N* = 95 (+/+), *N* = 118 (+/−), *N* = 35 (−/−).

The automated tracking of fish movements enables the calculation of the distance travelled by each fish (measured in pixels per minute) at 5 and 7 dpf under light and dark conditions. As shown in Fig. 4A, the distance travelled by 5 dpf heterozygous and homozygous mutants did not differ significantly from that of WT individuals under either condition. In contrast, at 7 dpf, the movement of heterozygous and homozygous mutants was significantly reduced in both light and dark conditions, compared to WT individuals (Fig. 4B). In all cases, locomotion was found to be greater in the dark, which is typically associated with increased anxiety due to the change in stimulus. These results indicate that *trip12* mutants have altered motor function. A similar outcome was observed in 5 dpf *trip12* morphants, which exhibited significantly diminished motility than their WT counterparts, regardless of light or dark conditions (Supplementary Fig. 3C).

### RNA sequencing

**Figure 5:**
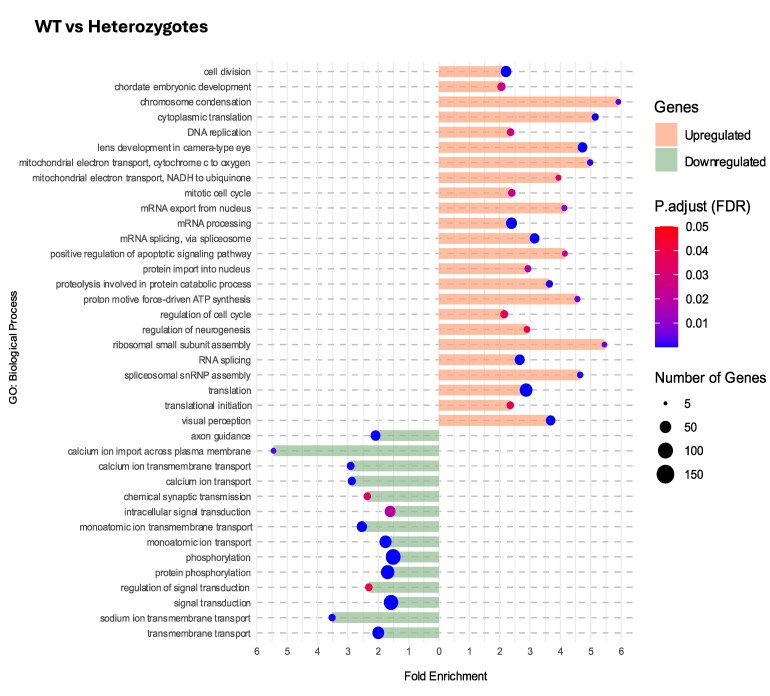
**Gene Ontology (GO) analysis for biological processes (BP).** Enriched BPs by upregulated (right) and downregulated (left) genes in *trip12* heterozygous (+/−) mutants compared to wildtype (WT, +/+). A detailed description of the analysis is provided in the ‘Materials and methods’ section. *N* = 96 (+/+), *N* = 121 (+/−). Each condition was polled in 3 biological replicates—3 pools of 32 WT individuals each; 2 pools of 40 each and 1 pool of 41 heterozygous individuals.

**Figure 6:**
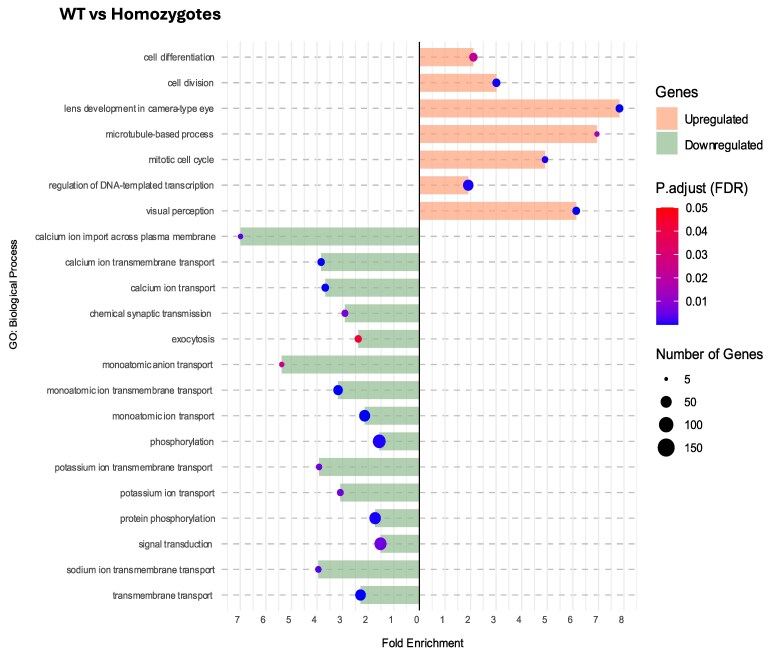
**Gene Ontology (GO) analysis for biological processes (BP).** Enriched BPs by upregulated (right) and downregulated (left) genes in *trip12* homozygous (−/−) mutants compared to wildtype (WT, +/+). A detailed description of the analysis is provided in the ‘Materials and methods’ section. *N* = 96 (+/+), *N* = 27 (−/−). Each condition was polled in 3 biological replicates—3 pools of 32 WT individuals each; 3 pools of 9 homozygous individuals each.

To explore the genes and molecular pathways affected by *trip12* knockout, we compared the transcriptomes of WT, *trip12* +/− and *trip12* −/− mutants. In the comparison between *trip12* +/+ and *trip12* +/−, we identified 4681 DEGs, with 1885 upregulated and 2796 downregulated in the *trip12* +/group. The comparison between *trip12* +/+ versus *trip12* −/− revealed 2671 DEGs, with 758 upregulated and 1913 downregulated in the *trip12* −/− group. A heat map of the expression of the top 50 DEGs is shown in Supplementary Fig. 4. GO analysis of the upregulated genes revealed enrichment for biological processes (BPs) related to general cellular functions such as cell cycle and gene expression regulation and developmental processes. Conversely, downregulated genes indicated a significant impact on BPs related to ion transport, signal transduction and, notably, synaptic transmission (Figs 5 and 6 and Supplementary Tables). Consistent with the altered BPs, enriched cellular components (CCs) included ribosomal and spliceosomal complexes and nuclear structures, whereas downregulated CCs predominantly clustered into neuronal components (Supplementary Fig. 5A and B and Supplementary Tables). Finally, upregulated molecular functions (MFs) mainly involve structural functions and nucleic acid binding activities, while downregulated MFs include ion, kinase and transferase activities (Supplementary Fig. 6A and B and Supplementary Tables). Specific enriched BPs, CCs and MFs related to the nervous system are summarized in Supplementary Figs 7 and 8.

**Figure 7:**
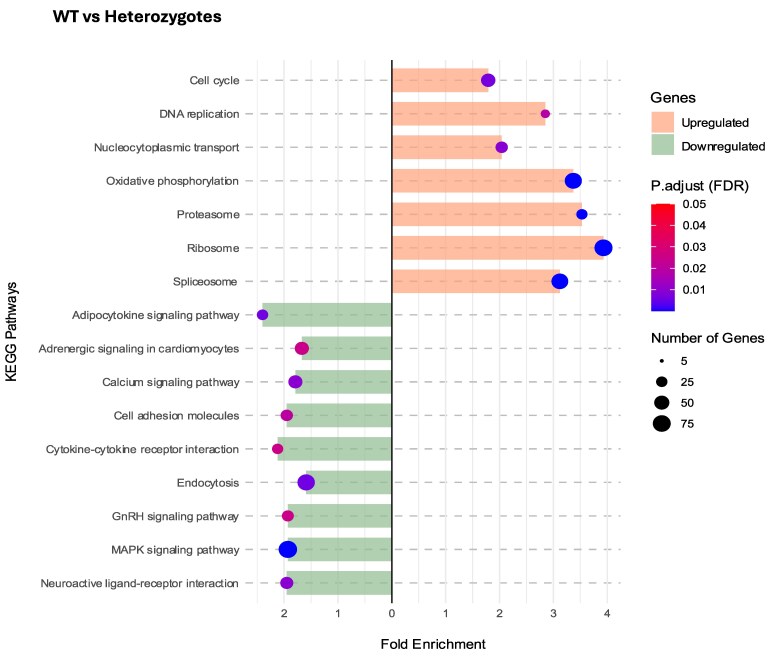
**KEGG analysis.** Enriched pathways by upregulated (right) and downregulated (left) genes in *trip12* heterozygous (+/−) mutants compared to wildtype (WT, +/+). A detailed description of the analysis is provided in the ‘Materials and methods’ section. *N* = 96 (+/+), *N* = 121 (+/−). Each condition was polled in 3 biological replicates—3 pools of 32 WT individuals each; 2 pools of 40 each and 1 pool of 41 heterozygous individuals.

**Figure 8:**
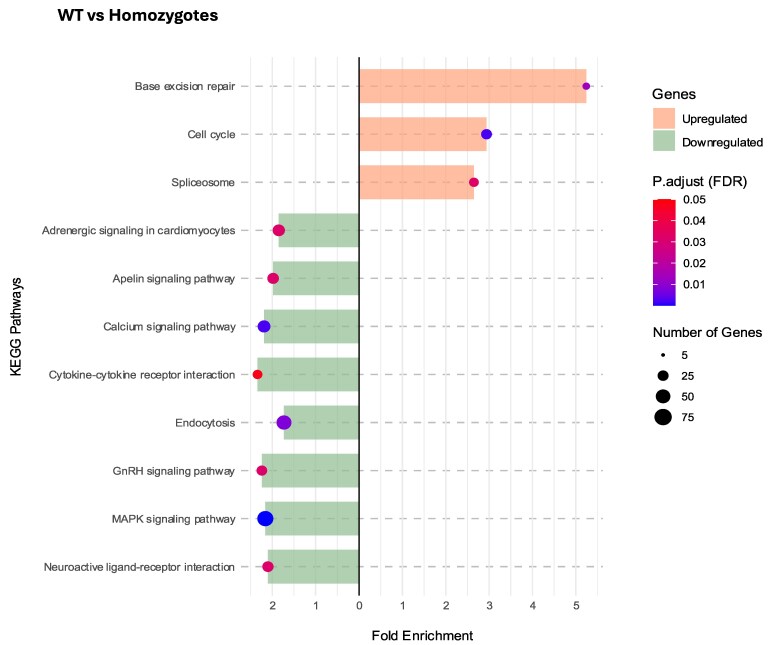
**KEGG analysis.** Enriched pathways by upregulated (right) and downregulated (left) genes in *trip12* homozygous (−/−) mutants compared to wildtype (WT, +/+). A detailed description of the analysis is provided in the ‘Materials and methods’ section. *N* = 96 (+/+), *N* = 27 (−/−). Each condition was polled in 3 biological replicates—3 pools of 32 WT individuals each; 3 pools of 9 homozygous individuals each.

To further investigate the functional implications of the DEGs in *trip12* +/− and *trip12* −/− individuals, we performed KEGG pathway analysis. Results for the upregulated genes (Figs 7 and 8) revealed enrichment for pathways related to cell cycle, DNA maintenance, nucleocytoplasmic transport and RNA and protein processing. Cellular energetics pathways were also enriched in *trip12 +/−* individuals, driven by upregulated genes involved in oxidative phosphorylation. Our analysis highlighted the influence of downregulated genes on key pathways (Figs 7 and 8), mainly related to cellular communication and signalling. These include endocytosis, adrenergic signalling in cardiomyocytes, calcium, GnRH and MAPK signalling pathways, as well as neuroactive ligand–receptor and cytokine–cytokine receptor interactions. Furthermore, adipocytokine signalling and cell adhesion molecules were enriched exclusively in *trip12 +/−* mutants, whereas apelin signalling was specific to *trip12 −/−* individuals.

## Discussion

In this study, we have successfully generated a CRISPR/Cas9 zebrafish mutant line *trip12*, a primary gene associated with ID. This mutant line recapitulates several phenotypical and motor abnormalities observed in patients with *TRIP12* mutations. While some *in vitro* and *in vivo* models have been previously generated,^4,16,38^ to our knowledge, this is the first fully characterized animal mutant model, with characterization at both the phenotypic and molecular levels in both heterozygous and homozygous states.

The generation of *trip12* CRISPR/Cas9 zebrafish mutants was confirmed by heteroduplex detection, and the introduction of premature stop codons was further validated by cloning, Sanger sequencing and *in silico* analysis (Fig. 1, Supplementary Figs 1 and 2). Although a heterozygous line was successfully maintained and bred, no adult homozygous mutants were identified, consistent with previous observations in *Trip12* homozygous mutant mice.^16^ Therefore, we performed a survival analysis from 0 hpf to determine the developmental stage at which homozygosity for the *trip12* mutation results in lethality. The results obtained demonstrate that homozygous mutants do not survive beyond 21 dpf, and heterozygous mutants exhibit a substantial mortality rate of 92% by 35 dpf, with only 8% of individuals reaching adulthood. These findings underscore the critical role of *trip12* in embryonic and larval development.

The phenotypic characterization of the mutants (Fig. 3) revealed abnormalities such as increased body length and eye area and decreased head diameter (microcephaly). These results are consistent with the dysmorphic features observed in nearly all patients with *TRIP12* mutations, with craniofacial and eye abnormalities being the most common.^3,7,39^ However, while our model exhibited increased body length, patients typically have normal stature, with only 17% presenting shorter stature.^39^ Analysis of morphants (Supplementary Fig. 3A and B) also showed a significant increase in body length and eye area, and 68% of morphants exhibited macrocephaly. While this differs from the microcephaly observed in mutants, macrocephaly has been reported in 13% of a cohort of patients with *TRIP12* variants.^39^

Given the lethality observed in homozygous zebrafish mutants and the diminished survival of heterozygous mutants, an evaluation of cardiac activity was conducted to determine whether heart function was responsible for the reduced survival, as this has been previously documented.^40^ It is also known that TRIP12 modulated the stability and proteolysis of SOX6, a process deemed essential for the sustenance of cardiac and skeletal muscle cells.^36^ Contrary to our initial hypothesis, our observations revealed that both heart gross morphology and cardiac rate were normal in heterozygous and homozygous mutants (Fig. 3D), indicating that cardiac failure was not the underlying cause of the observed abnormal survival. Subsequent analyses are currently being conducted to further explore the functional significance of *trip12* in the context of embryo and larval survival.

Abnormalities in motor functions, specifically motor delay and deficits in overall motor skill proficiency, have been reported in all cohorts of *TRIP12* patients and nearly in 90% of subjects. Therefore, the locomotor activity of morphant and mutant zebrafish was evaluated. While locomotion was similar in 5 dpf mutants (Fig. 4A) under both light and dark conditions, significantly lower activity was observed in 7 dpf heterozygous and homozygous mutants under both conditions (Fig. 4B). Similarly, 5 dpf morphants exhibited a marked decrease in locomotor activity, regardless of the light conditions (Supplementary Fig. 3C). The delayed onset motor disturbances in mutants, compared to morphants, may be explained by their *trip12* maternal contribution, as previously observed by Tseng *et al*.^41^ Overall, the zebrafish mutants recapitulate the motor abnormalities seen in patients.

Finally, to investigate the role of *trip12* at the transcriptomic and molecular level, we performed RNA sequencing. GO analysis revealed that upregulated genes are involved in a variety of BPs, suggesting multifunctional roles for *trip12* (Figs 5 and 6). Enriched CCs indicate involvement in various cellular structures (Supplementary Fig. 5), while MFs mainly reflect enzymatic activities alongside binding and structural functions (Supplementary Fig. 6).

The enrichment of BPs related to the cell cycle is consistent with the role of *TRIP12* in cell cycle progression,^1^ while terms related to development indicate the crucial role of *trip12* in embryogenesis. Other enriched terms highlight crucial roles for *trip12* in neurogenesis and related neuronal processes.

Among the KEGG pathways altered by overexpressed genes are proteasome, ribosome and spliceosome (Figs 7 and 8). This finding is consistent with the fact that alteration of E3 ubiquitin ligase activity can prevent the ubiquitination and subsequent degradation of substrates, leading to their accumulation and disrupted protein regulation.^42^ Consequently, this proteostasis imbalance may trigger compensatory activity of the proteasome and protein synthesis machinery in an attempt to restore homeostasis. In addition, ubiquitin signalling regulates RNA metabolism in the brain by controlling spliceosome remodelling, the levels of RNA-binding proteins and translation through ribosomal protein ubiquitination. Notably, disruptions in these pathways have been associated with neurodevelopmental disorders.^43^

Upregulated genes also enriched the oxidative phosphorylation pathway, essential for brain development and neuronal differentiation through energy production.^44-46^ This pathway generates reactive oxygen species, an excess of which leads to oxidative stress and potential damage to cellular components.^47,48^ The brain is particularly susceptible to these effects.^49,50^ Interestingly, excessive reactive oxygen species have been shown to negatively influence the proliferation of neuronal progenitor cells.^46^ In parallel, the cell cycle relies on TRIP12 to ensure its correct progression. This protein also regulates DNA replication timing during the S phase and coordinates mitotic entry and progression.^1^ Therefore, partial loss of *trip12* could allow residual ubiquitination activity, enabling limited cell-cycle progression and triggering compensatory upregulation of genes involved in DNA replication, nucleocytoplasmic transport and related synthesis pathways, as cells attempt to maintain proliferative capacity. In contrast, complete loss of *trip12* would result in cell-cycle arrest, triggering the activation of checkpoints and repair pathways such as base excision repair. Regarding the downregulated genes, they significantly enrich terms strongly associated with neurological processes, including BPs such as axon guidance and chemical synaptic transmission; MFs like calcium channel activity; and CCs such as axon membrane, neuron projection and synapse. Consequently, downregulated genes predominantly enrich KEGG pathways linked to neurodevelopment and neuronal communication, some of which are implicated in neurodevelopmental disorders like ID and ASD. These findings provide valuable insights into understanding Clark–Baraitser syndrome, which is primarily characterized by ID and commonly associated with ASD.^1^

In the context of neurodevelopment, the cell adhesion molecules pathway plays a pivotal role in the formation of neural circuits, contributing to axon guidance, neurogenesis, neuronal migration, synaptogenesis and myelination.^51-53^ Neuronal migration is fundamental to neurodevelopment, as it facilitates the spatial organization of neurons for optimal interactions.^54^ Similarly, calcium (Ca^2+^) release, acting as a second messenger, plays a vital role in neural precursor cell induction,^55^ synaptic activity,^56,57^ axon guidance, dendritic branching and neurotransmitter determination.^58^ Moreover, Ca^2+^ signalling has been associated with the pathobiology of epilepsy,^56^ potentially explaining why some patients with *TRIP12* mutations present seizures.^7,39^ Other critical pathways for CNS development and maturation are the MAPK signalling pathway,^59,60^ which regulates neuronal growth, proliferation and differentiation,^61,62^ and endocytosis, required for neuronal differentiation, migration, axon outgrowth and guidance.^63,64^

Beyond these neurodevelopmental processes, *trip12* mutants also exhibit dysregulated pathways that influence neuromodulation and synaptic communication. The GnRH signalling pathway exerts important neuromodulatory effects in many brain functions.^65^ Its involvement in preserving myelination and synaptic plasticity supports its contribution to cognitive function.^64^ Consistent with this, pulsed GnRH therapy has been shown to improve cognitive function in individuals with Down syndrome, thus highlighting that adequate GnRH signalling can positively impact intellectual performance.^66^ In line with this role in neuromodulation, the neuroactive ligand–receptor interaction pathway was also altered. This pathway, composed of neurotransmitter receptors and their ligands, regulates neurobiological functions such as synaptic transmission, neuronal communication and modulation of brain activity.^67,68^ Therefore, its dysregulation can significantly impact brain development, as evidenced by its involvement in neurodevelopmental disorders with cognitive impairments, supporting its potential contribution to intellectual disability.^68,69^ The cytokine–cytokine receptor interaction pathway has also been reported to be transcriptionally disrupted in other neurodevelopmental disorders,^69^ as cytokine signalling in the CNS participates in both development and homeostasis.^70^ Our transcriptomic analysis revealed significant dysregulation of genes in this pathway, with prominent involvement of TGF-β family members, which are heavily associated with neuronal development and neurological disorders due to their role in regulating cell fate determination and patterning in the CNS.^71^

Finally, the adipocytokine signalling pathway, implicated in energy homeostasis^72,73^ and enriched in *trip12 +/−* mutants, may provide a plausible explanation for the obesity observed in some patients with *TRIP12* mutations.^7^ In addition to its metabolic role, this pathway influences neurological processes such as neurogenesis and synaptogenesis.^74^ Although only significantly enriched by downregulated genes in *trip12 −/−*, apelin, a multifaceted protein, plays a crucial role in neuronal structure and function, oxidative stress and Ca^2+^ signalling.^75^ Therefore, the downregulation of the apelin signalling pathway may contribute to neurodevelopmental disorders.

Overall, this study provides a comprehensive explanation on how disruption of *trip12* alters gene expression, elucidating its role in shaping the structure and function of the nervous system, among other processes. Notably, this is the first in-depth study to unravel the intricate molecular dynamics resulting from the *trip12* mutation, potentially explaining the alterations observed in patients with mutations in this gene.

## Supplementary Material

fcag276_Supplementary_Data

## Data Availability

Data will be made available by the corresponding author upon reasonable request.
