# Supplementary material for: Unraveling the impact of trip12 on neurodevelopment: insights from a zebrafish model
Source: Brain Commun. 2026 Jul 15;8(4):fcag276. doi: 10.1093/braincomms/fcag276 (PMC13397127; doi:10.1093/braincomms/fcag276)
Supplement: fcag276_Supplementary_Data [file fcag276_supplementary_data.zip › Supplementary_material_APL-MRS.docx]

**Unraveling the impact of *trip12* on neurodevelopment: insights from a zebrafish model**

Maider Roibás-Santos, Paula Suárez-Bregua, Josep Rotllant, Ángel Carracedo, Catarina Allegue, Laura Sánchez, Andrés Blanco-Hortas, and Alba Pensado-López

**Supplementary Material**

**Methods**

**Antisense Morpholino Oligonucleotide injection and *trip12* knockdown verification**

An antisense morpholino oligonucleotide (MO) targeting the translation initiation site of *trip12* was purchased to GeneTools, LLC (*trip12*-MO_5’- GGACATTGGCACCTCTCTCCTGAAG -3’). The MO was resuspended in water to a concentration of 1mM, and approximately 5 nl was micro-injected into one-cell stage zebrafish embryos. To verify the efficacy of *trip12* knockdown, the expression of *ptf1a,* a known target of *trip12* ^1,2^*,* was analyzed by quantitative PCR (qPCR). Total RNA was isolated from 20 embryos at 48 hpf using the miRNeasy Micro Kit (Qiagen), and RNA concentration was measured using a NanoDrop® 2000 spectrophotometer (Thermo Fisher Scientific). cDNA was synthesized using the AffinityScript Multiple Temperature cDNA Synthesis kit (Agilent), and qPCR was performed using the Brilliant III Ultra-Fast SYBR Green QPCR Master Mix kit (Agilent) on a Stratagene Mx3005P system (Agilent). The following primers were used for *ptf1a*: F_5’-CCACACAGTGACGCGTTAAA-3’ and R_5’-AGAGAGTGTCCTGCGAGAGG -3’. *β-actin* (F_5’-TGAAATTGCCGCACTGGTTG-3’; R_5’-TACCAACCATGACACCCTGATG-3’) was used as the reference gene for quantitative analysis. Results were expressed as fold change relative to the control using the ΔΔCt method. Data were analyzed using GraphPad Prism version 7.02. Statistical comparisons were performed using an unpaired T-test. Statistically significant differences were defined as *P* < 0.05. In figures, levels of significance are represented as follows: * *P* < 0.05; ** *P* < 0.01, *** *P* < 0.001, **** *P* < 0.0001.

**Morphant phenotyping and locomotion characterization**

Embryos at 48 hpf were anesthetized with 0.02% tricaine methanesulfonate (MS-222, Sigma-Aldrich), and images were captured using a Nikon Ds-Ri1 camera attached to an inverted fluorescence microscope (AZ100 Multizoom, Nikon). Images were analyzed using Nis-Elements BR (Version 4.13.04 64-bit) to measure three phenotypic features: body length, head diameter, and eye area (Figure 3A). General motor function was evaluated in 5 dpf larvae as described in the corresponding section of the main manuscript. Measurement data were exported to Microsoft Excel and analyzed using GraphPad Prism version 7.02. Statistical comparisons were performed using unpaired T-test. Statistically significant differences were defined as *P* < 0.05. In figures, levels of significance are represented as follows: * *P* < 0.05; ** *P* < 0.01, *** *P* < 0.001, **** *P* < 0.0001.

**Results**

**MO injections produce *trip12* knockdown**

The evaluation of the expression of *ptf1a* by qPCR revealed that morphants have increased expression of this gene compared to their WT siblings (Supplementary Figure 2D). These results confirm that the MO-mediated knockdown of *trip12* was successful. It has been previously reported that TRIP12 ubiquitinates PTF1a, promoting its degradation, and that mutations in *trip12* lead to *ptf1a* upregulation^1^. PTF1a is required for the specification of inhibitory neurons, and, together with *atoh1*, it is essential for the specification of glutamatergic neurons and Purkinje cells in zebrafish^3,4^. Thus, TRIP12 may regulate neuronal specification via PTF1a degradation.

***trip12* morphants display phenotypic and locomotor alterations**

Morphants at 48 hpf were imaged and analyzed. Similar to the observations in mutants (Figure 2), the general phenotype of morphants appeared normal compared to WT individuals, but measurements of body length, head diameter, and eye area revealed statistically significant differences. As shown in Supplementary Figure 2A, morphants exhibited increased body length (+/+: 1440.14 µm; MO: 1542.6 µm) and eye area (+/+: 7262.57 µm^2^; MO: 8181.95 µm^2^), as well as increased head diameter (+/+: 199.62 µm; MO: 206.1 µm). In summary, 83% of morphants had increased body length (Supplementary Fig. 2B, left panel), 68% had increased head diameter (Supplementary Figure 2B, central panel), and 83% of morphants had increased eye area (Supplementary Figure 2C, right panel). The dysmorphic features observed in morphants are consistent with the phenotypic abnormalities typically observed in patients with *TRIP12* mutations^5–7^.

Regarding locomotor function, morphants displayed decreased activity, measured as pixels moved per minute, under both light and dark conditions (Supplementary Figure 3C), similar to what was observed in heterozygous and homozygous mutants (Figure 4). These results are consistent with the observations in patients with *TRIP12* variants, who present persistent motor problems, specially concerning fine motor skills, in more than 70% of the cases^6,8^.

**Supplementary Figures**


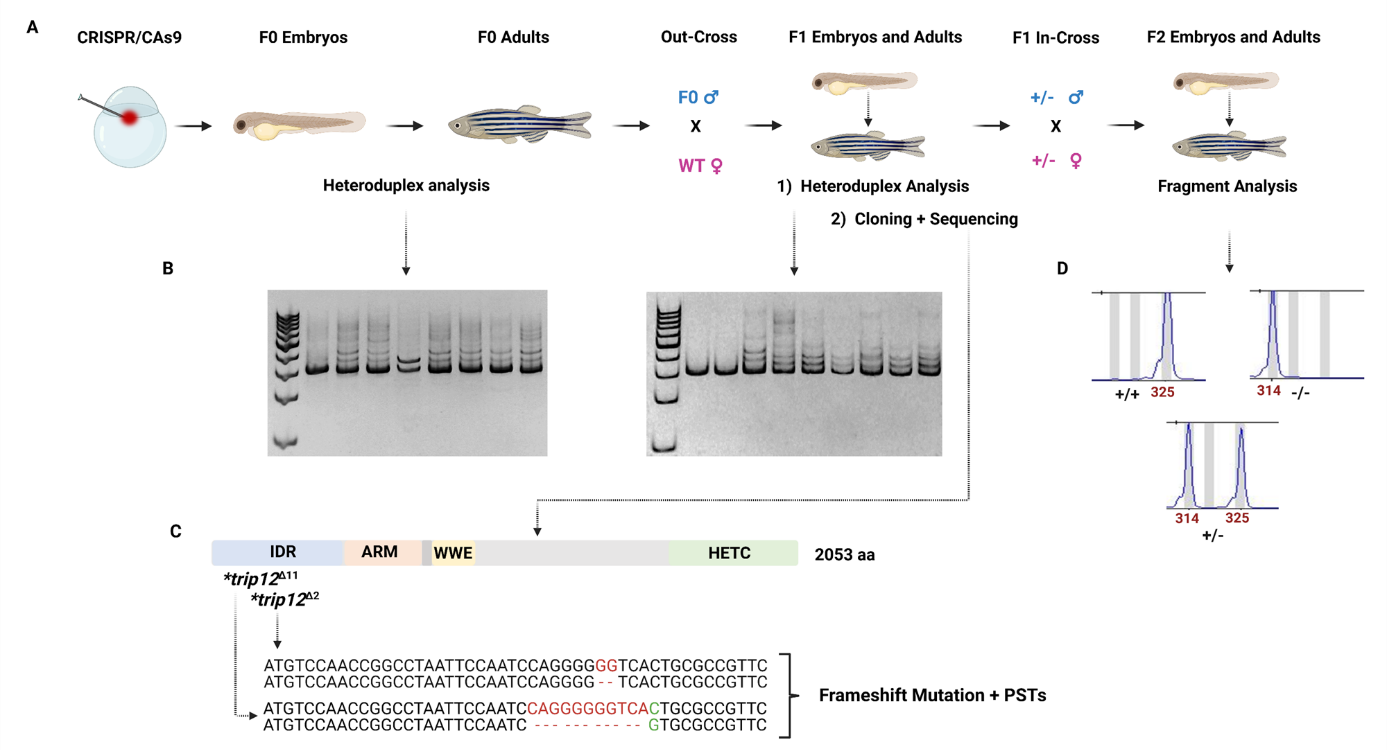


**Supplementary Figure 1. CRISPR/Cas9 *trip12* knockout**. **A**) Schematic representation of CRISPR/Cas9 *trip12* knockout and genotype characterization. **B**) Representative images of heteroduplex analysis for F0 adults (left) and F1 individuals (right). **C**) Schematic representation of the 2053 aa Trip12 protein and mutant sequence of exon 1 resulting from CRISPR/Cas9 knockout. **D**) Example of results obtained from fragment analysis of F2 wildtype (WT, +/+), heterozygous (+/-) and homozygous (-/-) individuals. For CRISPR/Cas injections, N=150. Uncropped gels are shown in Supplementary Figure 2.


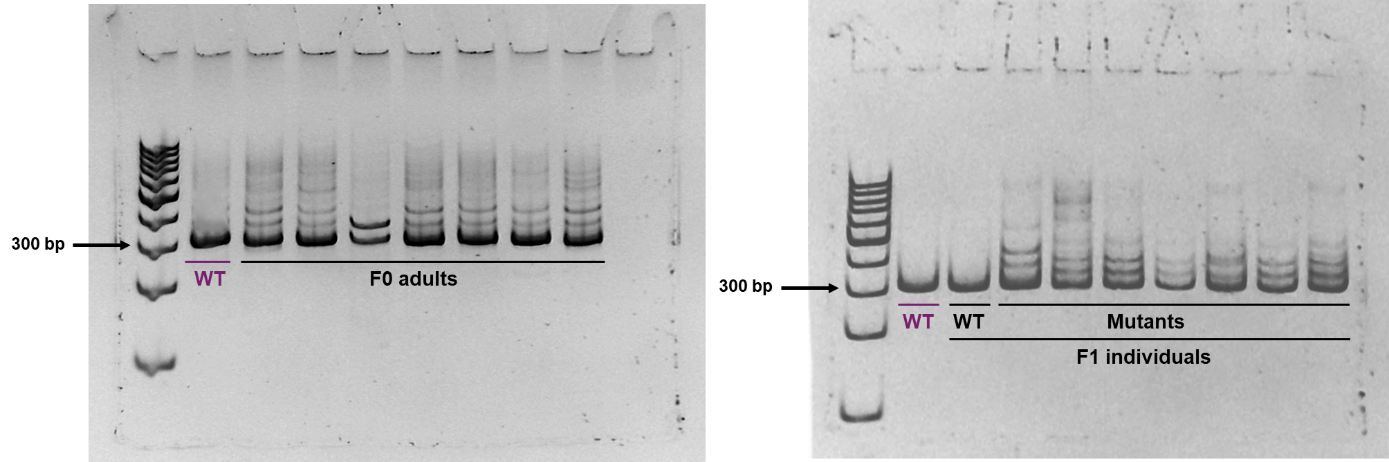


**Supplementary Figure 2. Uncropped gels shown in Supplementary Fig 1B**. Representative images of heteroduplex analysis for F0 adults (left). Lane 1, 100-1000 base pair (bp) ladder; Lane 2, control WT; Lanes 3-9, F0 mutants. Representative images of heteroduplex analysis for F1 individuals (right). Lane 1, 100-1000 base pair ladder (bp); Lane 2, control WT; Lane 3, F1 WT; Lanes 4-10, F1 mutants.


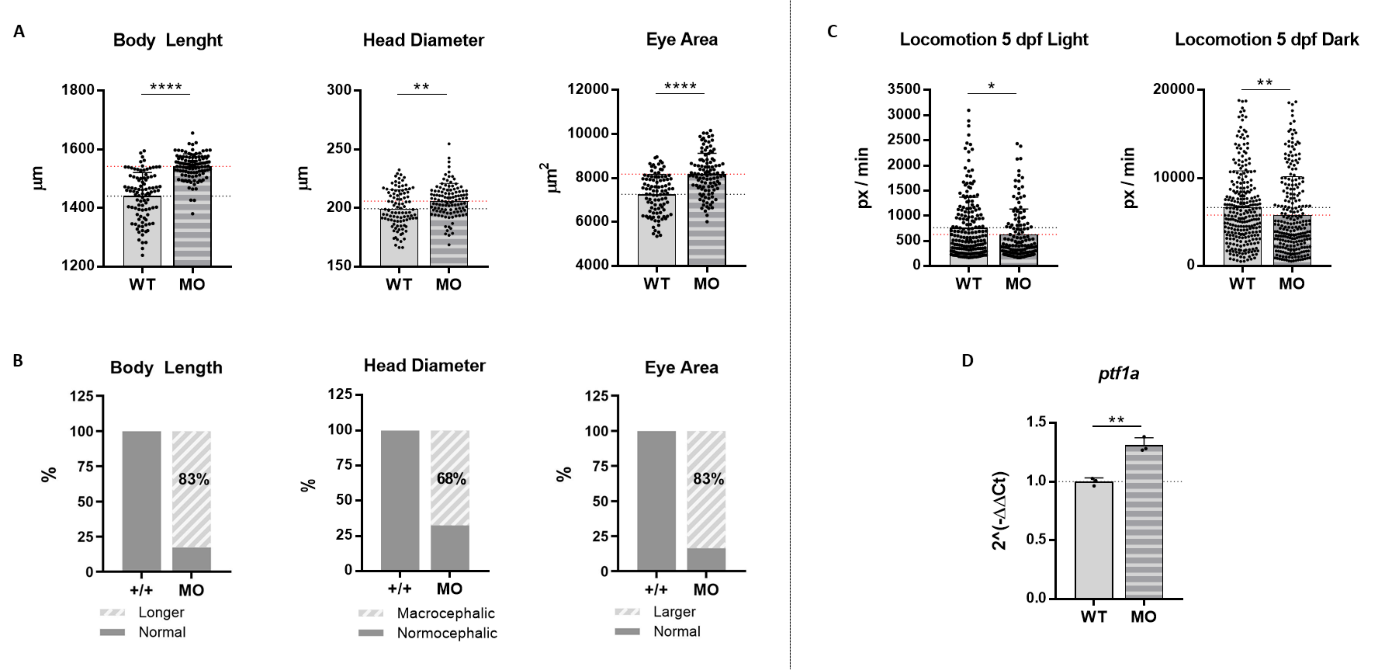


**Supplementary Figure 3. Phenotype and locomotion analysis of *trip12* morphants**. **A**) Body length, head diameter and eye area comparison among wildtype (WT) and morphants (MO). **B**) Percentage of morphants presenting longer body length, macrocephaly and larger eye area. **C**) Locomotor analysis of *trip12* morphants. Total count of pixels moved per minute in light and dark conditions for 5 dpf individuals. **D**) Relative expression of *ptf1a* in *trip12* morphants. Each dot represents individual larvae. Statistical comparisons were performed using unpaired T-test and statistically significant differences were defined as p < 0.05. Levels of significance are represented as follows: * p < 0.05; ** p < 0.01, *** p < 0.001, **** p < 0.0001. For phenotype characterization, N= 100 (WT), N= 109 (MO). For locomotion analysis, N= 256 (WT), N= 251 (MO).

**
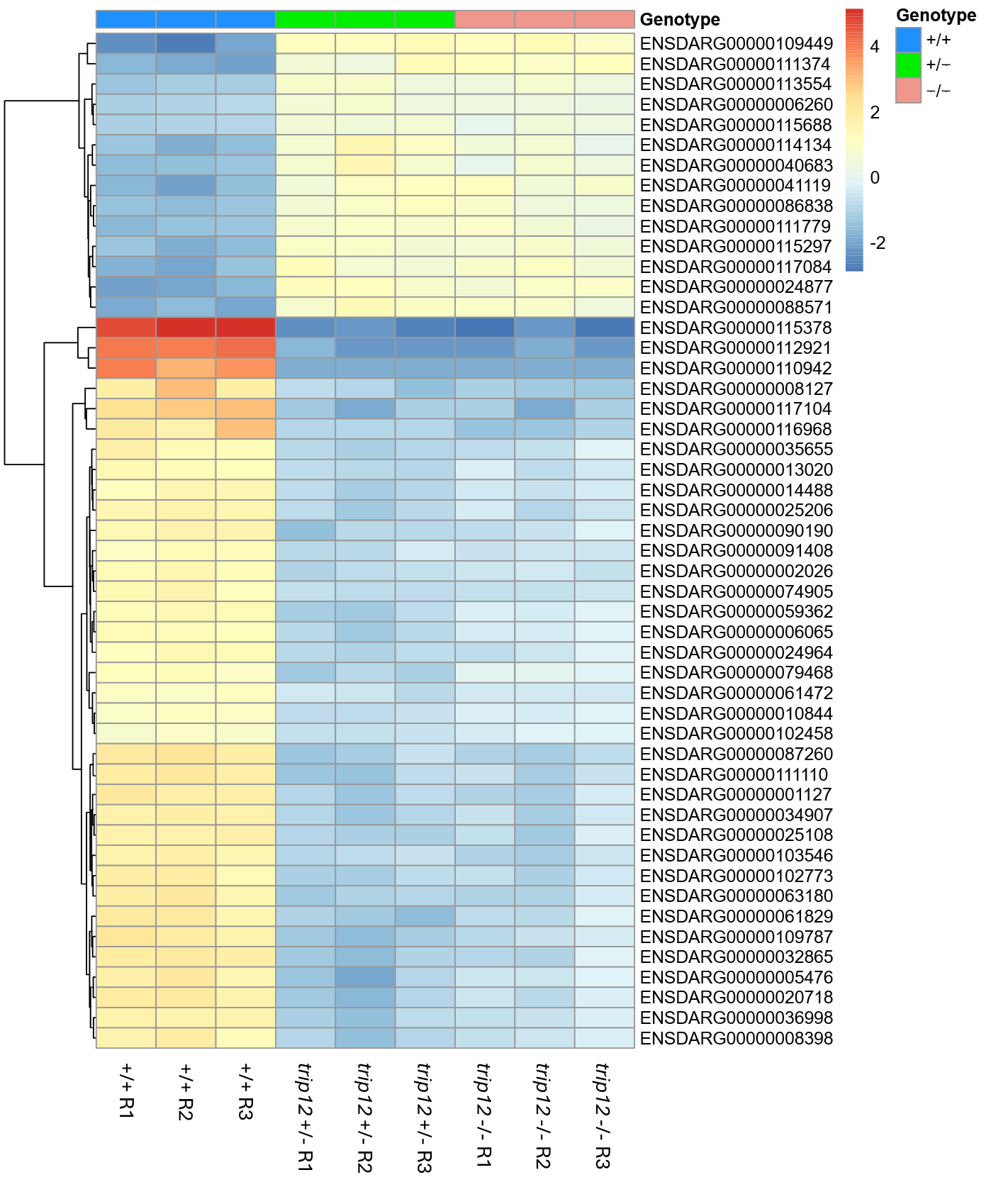
**

**Supplementary Figure 4. Heat map of the expression of the top 50 DEGs.** Comparison of gene expression among wildtype (WT, +/+), *trip12* heterozygous (+/-) and *trip12* homozygous (-/-) mutants, by replicates. A detailed description of the analysis is provided in the main manuscript. N= 96 (+/+), N= 121 (+/-), N= 27 (-/-). Each condition was polled in 3 biological replicates - 3 pools of 32 WT individuals each; 2 pools of 40 each and 1 pool of 41 heterozygous individuals; 3 pools of 9 homozygous individuals each.

**
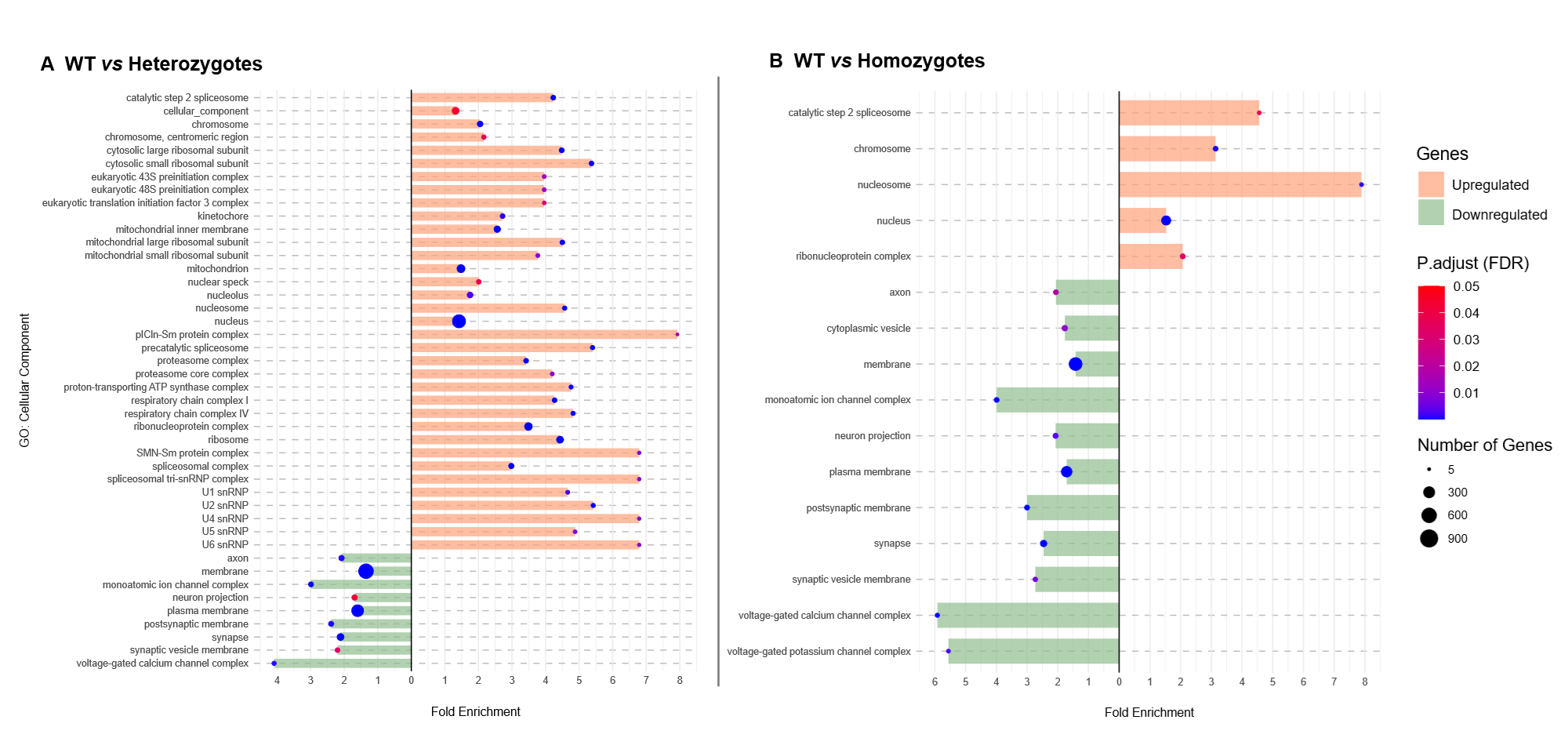
**

**Supplementary Figure 5. Go Ontology (GO) analysis for Cellular Components (CCs).** Enriched CCs of upregulated (orange) and downregulated (green) genes in *trip12* heterozygous (+/-) (**A**) and *trip12* homozygous (-/-) (**B**) mutants compared to wildtype (WT, +/+). A detailed description of the analysis is provided in the main manuscript. N= 96 (+/+), N= 121 (+/-), N= 27 (-/-). Each condition was polled in 3 biological replicates - 3 pools of 32 WT individuals each; 2 pools of 40 each and 1 pool of 41 heterozygous individuals; 3 pools of 9 homozygous individuals each.

**
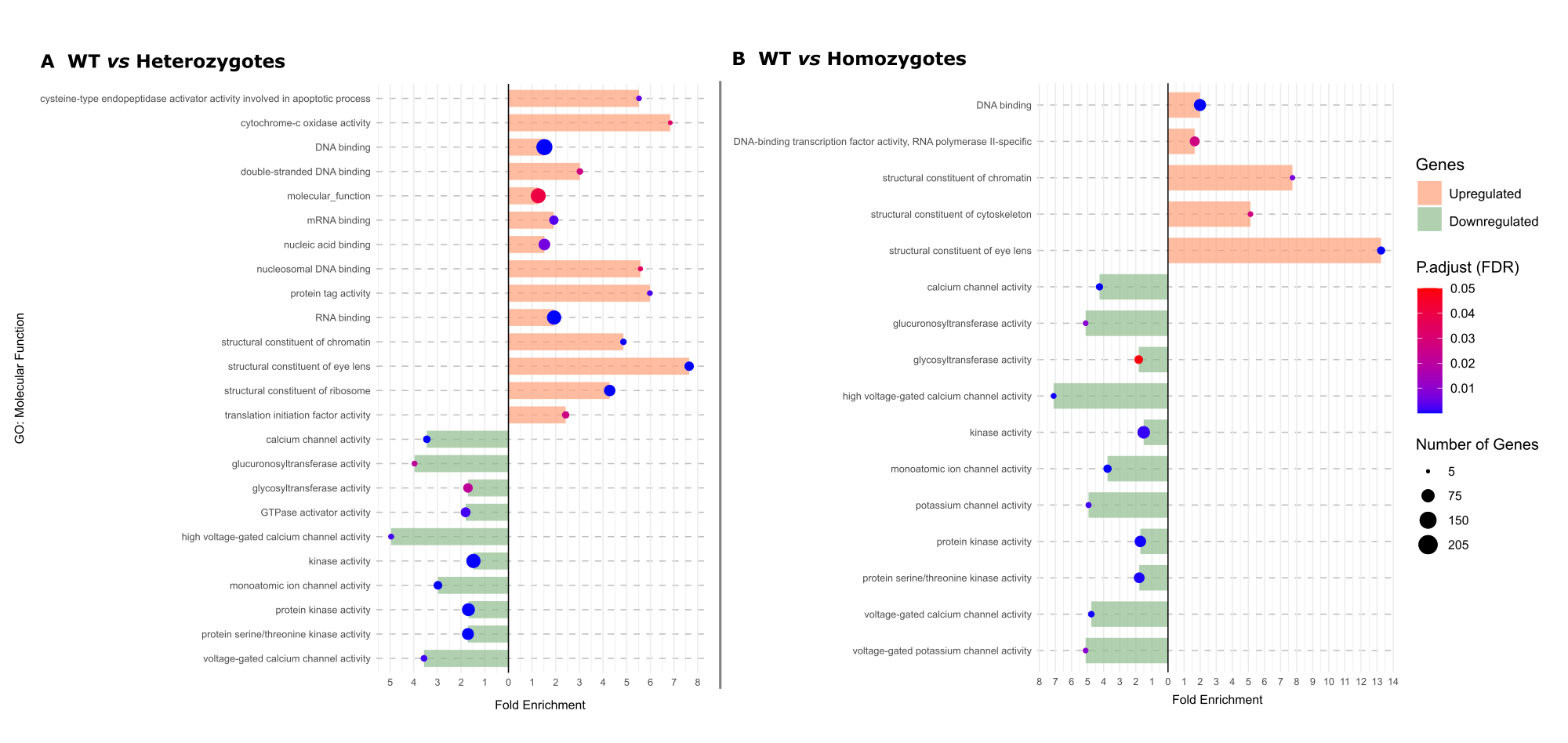
**

**Supplementary Figure 6. Go Ontology (GO) analysis for Molecular Functions (MFs).** Enriched MFs of upregulated (orange) and downregulated (green) genes in *trip12* heterozygous (+/-) (**A**) and *trip12* homozygous (-/-) (**B**) mutants compared to wildtype (WT, +/+). A detailed description of the analysis is provided in the main manuscript. N= 96 (+/+), N= 121 (+/-), N= 27 (-/-). Each condition was polled in 3 biological replicates - 3 pools of 32 WT individuals each; 2 pools of 40 each and 1 pool of 41 heterozygous individuals; 3 pools of 9 homozygous individuals each.


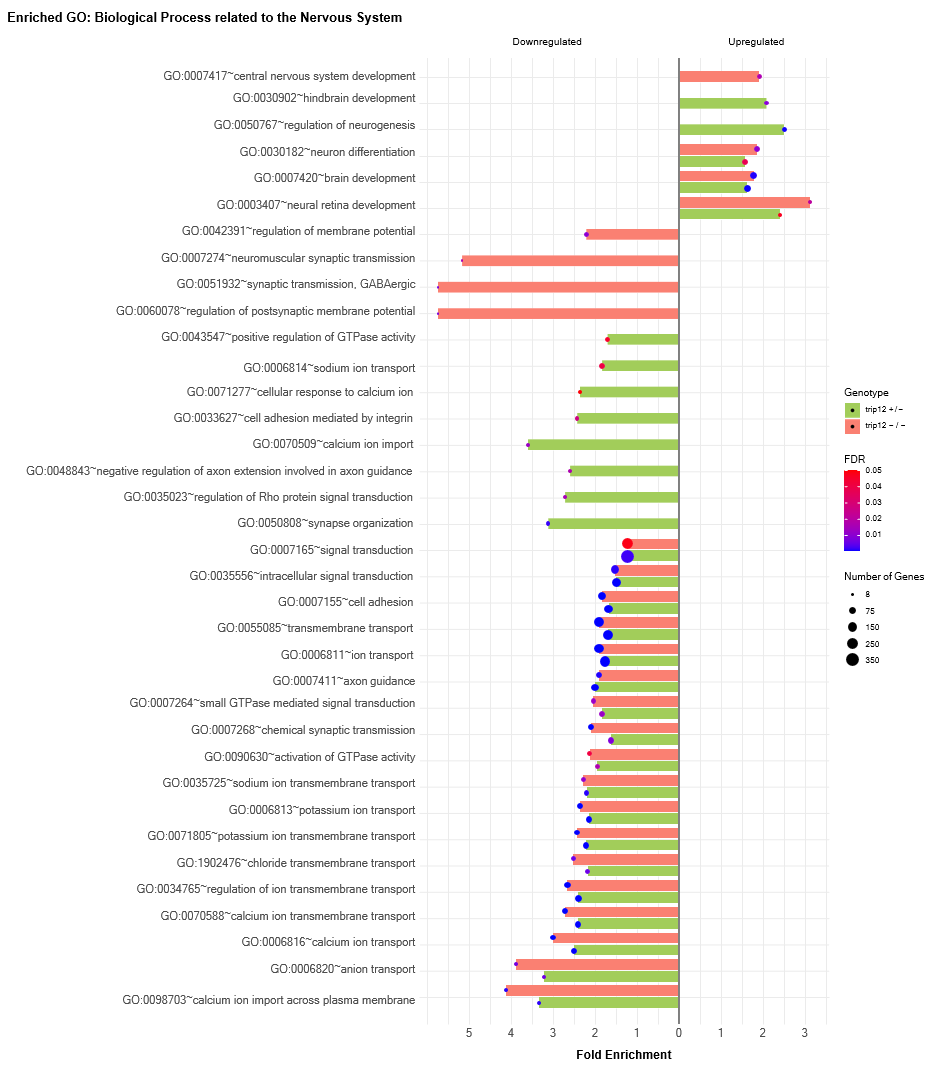


**Supplementary Figure 7. Gene Ontology (GO) analysis for Biological Process (BPs) related to the Nervous System.** Enriched BPs by downregulated and upregulated genes in *trip12* heterozygous (+/-, green) and *trip12* homozygous (-/-, red). A detailed description of the analysis is provided in the main manuscript. N= 96 (+/+), N= 121 (+/-), N= 27 (-/-). Each condition was polled in 3 biological replicates - 3 pools of 32 WT individuals each; 2 pools of 40 each and 1 pool of 41 heterozygous individuals; 3 pools of 9 homozygous individuals each.

**
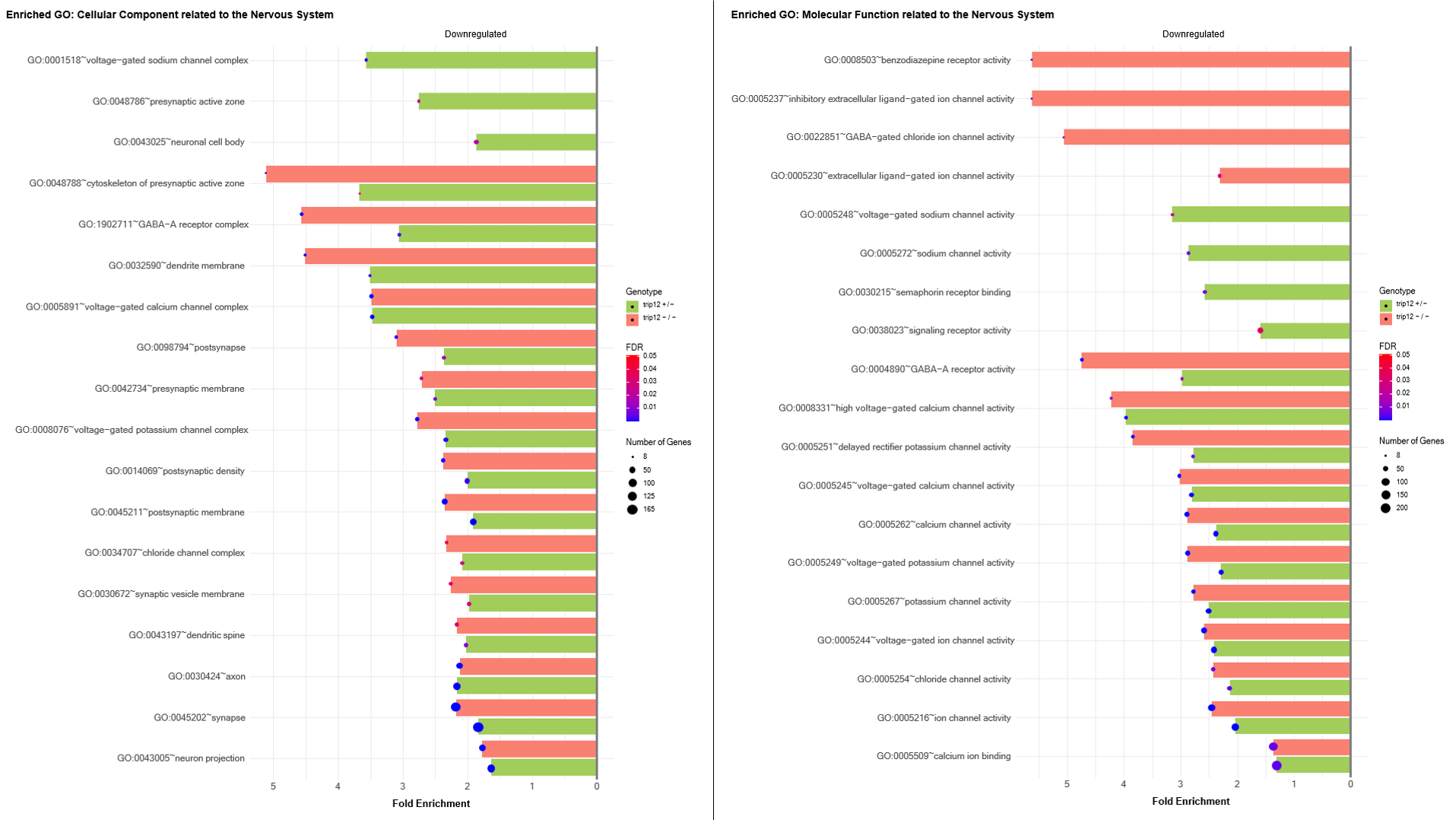
**

**Supplementary Figure 8. Gene Ontology (GO) analysis for Cellular Components (CCs, left) and Molecular Functions (MFs), related to the Nervous System.** Enriched CCs and MFs by downregulated genes in *trip12* heterozygous (+/-, green) and *trip12* homozygous (-/-, red). A detailed description of the analysis is provided in the main manuscript. N= 96 (+/+), N= 121 (+/-), N= 27 (-/-). Each condition was polled in 3 biological replicates - 3 pools of 32 WT individuals each; 2 pools of 40 each and 1 pool of 41 heterozygous individuals; 3 pools of 9 homozygous individuals each.

**Supplementary References**

1. Hanoun N, Fritsch S, Gayet O, et al. The E3 Ubiquitin Ligase Thyroid Hormone Receptor-interacting Protein 12 Targets Pancreas Transcription Factor 1a for Proteasomal Degradation. *Journal of Biological Chemistry*. 2014;289(51):35593-35604. doi:10.1074/jbc.M114.620104

2. Brunet M, Vargas C, Larrieu D, Torrisani J, Dufresne M. E3 Ubiquitin Ligase TRIP12: Regulation, Structure, and Physiopathological Functions. *IJMS*. 2020;21(22):8515. doi:10.3390/ijms21228515

3. Kani S, Bae YK, Shimizu T, et al. Proneural gene-linked neurogenesis in zebrafish cerebellum. *Dev Biol*. 2010;343(1-2):1-17. doi:10.1016/j.ydbio.2010.03.024

4. Bae YK, Kani S, Shimizu T, et al. Anatomy of zebrafish cerebellum and screen for mutations affecting its development. *Dev Biol*. 2009;330(2):406-426. doi:10.1016/j.ydbio.2009.04.013

5. Donoghue T, Garrity L, Ziolkowski A, McPhillips M, Buckman M, Goel H. Novel de novo TRIP12 mutation reveals variable phenotypic presentation while emphasizing core features of TRIP12 variations. *Am J Med Genet A*. 2020;182(7):1801-1806. doi:10.1002/ajmg.a.61618

6. Aerden M, Denommé-Pichon AS, Bonneau D, et al. The neurodevelopmental and facial phenotype in individuals with a TRIP12 variant. *Eur J Hum Genet*. 2023;31(4):461-468. doi:10.1038/s41431-023-01307-x

7. Kondo Y, Aoyama K, Suzuki H, et al. De novo 2q36.3q37.1 deletion encompassing TRIP12 and NPPC yields distinct phenotypes. *Hum Genome Var*. 2020;7:19. doi:10.1038/s41439-020-0107-1

8. Zhang J, Gambin T, Yuan B, et al. Haploinsufficiency of the E3 ubiquitin-protein ligase gene TRIP12 causes intellectual disability with or without autism spectrum disorders, speech delay, and dysmorphic features. *Hum Genet*. 2017;136(4):377-386. doi:10.1007/s00439-017-1763-1
